# APSIC Guidelines for environmental cleaning and decontamination

**DOI:** 10.1186/s13756-015-0099-7

**Published:** 2015-12-29

**Authors:** Moi Lin Ling, Anucha Apisarnthanarak, Le Thi Anh Thu, Victoria Villanueva, Costy Pandjaitan, Mohamad Yasim Yusof

**Affiliations:** Singapore General Hospital, Outram Road, 169608 Singapore; Thammasat University Hospital, Khlong Luang District, Thailand; Cho Ray Hospital, Hồ Chí Minh, Vietnam; Chong Hua Hospital, Cebu City, Philippines; Association of Infection Prevention Control Nurse, Jakarta, Indonesia; University of Malaya Medical Centre, Kuala Lumpur, Malaysia

**Keywords:** Environmental cleaning, Decontamination, Environmental hygiene

## Abstract

This document is an executive summary of APSIC Guidelines for Environmental Cleaning and Decontamination. It describes best practices in routine cleaning and decontamination in healthcare facilities as well as in specific settings e.g. management of patients with isolation precautions, food preparation areas, construction and renovation, and following a flood. It recommends the implementation of environmental hygiene program to keep the environment safe for patients, staff and visitors visiting a healthcare facility. Objective assessment of cleanliness and quality is an essential component of this program as a method for identifying quality improvement opportunities. Recommendations for safe handling of linen and bedding; as well as occupational health and safety issues are included in the guidelines. A training program is vital to ensure consistent adherence to best practices.

## Background

Contamination of hospital equipment, medicines, and water supplies with hospital pathogens is a well-recognized cause of common-source outbreaks of infection [1–9]. The intent of this document is to highlight practical recommendations in a concise format designed to assist healthcare facilities at Asia Pacific region in implementing an environmental hygiene program.

This document is a summary of the APSIC Guidelines For Environmental Cleaning And Decontamination developed by the Asia Pacific Society of Infection Control (APSIC).

## Review

### Workgroup composition

APSIC convened Infection Prevention and Control experts from Asia Pacific region to develop the APSIC Guidelines For Environmental Cleaning And Decontamination. The members of this workgroup are the authors of this paper.

### Literature review and analysis

For the APSIC guideline, the workgroup reviewed previously published guidelines and recommendations relevant to each section and performed computerized literature searches using PubMed.

### Process

The workgroup met on 2 occasions as well as discussed via email correspondences to complete the development of the guideline. Criteria for grading the strength of recommendation and quality of evidence are described in Table 1. The draft was then submitted to APSIC Executive Committee and national Infection Control societies in Asia Pacific. Comments obtained were then reviewed by the workgroup for necessary edits, following which the final copy was circulated for approval and endorsement by the APSIC Executive Committee and national societies from the Asia Pacific region.

**Table 1:** Categories for strength of each recommendation

Categories for strength of each recommendation	
CATEGORY	DEFINITION
A	Good evidence to support a recommendation for use
B	Moderate evidence to support a recommendation for use.
C	Insufficient evidence to support a recommendation for or against use
D	Moderate evidence to support a recommendation against use.
E	Good evidence to support a recommendation against use.
Categories for quality of evidence on which recommendations are made	
GRADE	DEFINITION
I	Evidence from at least one properly randomized, controlled trial.
II	Evidence from at least one well-designed clinical trial without randomization, from cohort or case-controlled analytic studies, preferably from more than one centre, from multiple time series, or from dramatic results in uncontrolled experiments.
III	Evidence from opinions of respected authorities on the basis of clinical experience, descriptive studies, or reports of expert committees.

### General cleaning practices

Health care settings comprised areas that require either Hotel Clean or Hospital Clean based on the risk of the patient population in the area. Hotel Clean is a measure of cleanliness based on visual appearance that includes dust and dirt removal, waste disposal and cleaning of windows and surfaces. In addition to routine cleaning, additional cleaning practices and/or the use of personal protective equipment for cleaning may be required in health care settings under special circumstances. Hotel Clean is the basic cleaning that takes place in all areas of a health care setting [2].

Hospital Clean is a measure of cleanliness routinely maintained in care areas of the health care setting. Hospital Clean is ‘*Hotel Clean*’ with the addition of disinfection, increased frequency of cleaning; auditing and other infection control measures in client/patient/resident care areas [2].

### Components of hotel clean

- Floors and baseboards are free of stains, visible dust, spills and streaks
- Walls, ceilings and doors are free of visible dust, gross soil, streaks, spider webs and handprints
- All horizontal surfaces are free of visible dust or streaks (includes furniture, window ledges, overhead lights, phones, picture frames, carpets etc.)
- Bathroom fixtures including toilets, sinks, tubs and showers are free of streaks, soil, stains and soap scum
- Mirrors and windows are free of dust and streaks
- Dispensers are free of dust, soiling and residue and replaced/replenished when empty
- Appliances are free of dust, soiling and stains
- Waste is disposed of appropriately
- Items that are broken, torn, cracked or malfunctioning are replaced

### Cleaning best practices at patient care areas [10–14]

Housekeeping in the health care setting should be performed on a routine and consistent basis to provide for a safe and sanitary environment. Maintaining a clean and safe health care environment is an important component of infection prevention and control. The frequency of cleaning and disinfecting individual items or surfaces in a particular area or department depends on:

- Whether surfaces are high-touch or low-touch;
- The type of activity taking place in the area and the risk of infection associated with it (e.g., critical care areas vs. meeting room);
- The vulnerability of patients housed in the area; and
- The probability of contamination based on the amount of body fluid contamination surfaces in the area might have or be expected to have

See example in Table 2.

**Table 2:** Risk Stratification Matrix to determine the frequency of cleaning (example)

Discipline	Probability of contamination	Potential for Exposure	Population	Total score
	Light :1	High-touch : 3	Less susceptible:0	
	Moderate: 2	Low -touch:1	More susceptible: 1	
	Heavy: 3			
Renal	2	3	1	6
Burns	2	3	1	6
Respiratory Medicine	2	3	0	5
General Medicine	2	3	1	6
Colorectal Surgery	2	3	0	5
Oncology	2	3	1	6
Neurology and Neurosurgery	2	3	0	5
Surgical Intensive Care Unit	3	3	1	7
Medical Intensive Care Unit	3	3	1	7

Interpretation of total score: **7**: **High risk**: clean after each case/event/procedure and at least twice per day, clean additionally as required; **4**–**6**: **Moderate Risk** : clean at least once daily, clean additionally as required (e.g. Gross soiling), **2**–**3** : **Low risk**: clean according to a fixed scheduled, clean additionally as required (e.g. Gross soiling)

### Recommendations

1. Housekeeping in the health care setting should be performed on a routine and consistent basis to provide for a safe and sanitary environment. [BIII]
2. Adequate resources must be devoted to Housekeeping Department in all health care settings that include:
  - Single individual with assigned responsibility for the care of the physical facility;
  - Written procedures for cleaning and disinfection of care areas and equipment that include:
    i. Defined responsibility for specific items and areas;
    ii. Procedures for daily and terminal cleaning;
    iii. Procedures for cleaning in construction/renovation areas;
    iv. Procedures for cleaning and disinfecting areas contaminated with VRE and C. difficile;
    v. Procedures for outbreak management;
    vi. Cleaning standards and frequency;
  - Adequate human resources to allow thorough and timely cleaning and disinfection;
  - Education and continuing education of cleaning staff;
  - Monitoring of environmental cleanliness; and
  - Ongoing review of procedures. [BII]
3. If housekeeping services are contracted out, the Occupational Health and Safety policies of the contracting services must be consistent with the facility’s Occupational Health and Safety policies. [BII]
4. Housekeeping Department staffing levels should reflect the physical nature and the acuity of the facility; levels of supervisory staff should be appropriate to the number of staff involved in cleaning. [BIII]
5. Non-critical medical equipment requires cleaning and disinfection after each use. [AII]
6. Each health care setting should have written policies and procedures for the appropriate cleaning of non-critical medical equipment that clearly defines the frequency and level of cleaning and which assigns responsibility for the cleaning. [BIII]
7. Cleaning schedules should be developed, with frequency of cleaning reflecting whether surfaces are high-touch or low-touch, the type of activity taking place in the area and the infection risk associated with it; the vulnerability of the patients housed in the area; and the probability of contamination. [BIII]
8. All environmental service staff entering a room, which is on Contact Precautions, must put on a gown and gloves on entering the room and must remove them and perform hand hygiene on leaving the room. [BII]
9. For adequate removal of C. difficile, the use of a sporicidal agent for disinfection after the room has been cleaned is needed. [BII]
10. Housekeeping staff entering a room with Airborne Precautions must wear a fit tested N95 respirator. [BII]
11. Housekeeping and clinical staff entering a room with Droplet Precautions must wear a surgical mask. [BII]

### Cleaning spills of blood and body substances

#### Recommendations

1. Spills of blood and other bodily substances must be contained, cleaned and the area disinfected immediately. [BII]
2. Absorbent disinfectant spillage granules may be more convenient to use instead of liquid disinfectant. [BIII]

### Infection control during construction and renovation

Construction and renovation activities in the hospital may be associated with transmission of pathogens such as filamentous fungi, including *Aspergillus spp*, *Candida spp*, *Fusarium* and also bacteria such as *Legionella* and *Nocardia* [15, 16]. The most commonly reported hospital construction-related infection is *Aspergillus*, which represent the greatest threat to neutropenic patients.

‘*Construction Clean*’ is the level of cleaning performed by construction workers to remove gross soil, dust and dirt, construction materials and workplace hazards within the construction zone. This is done at the end of the day, or more frequently if needed, to avoid accumulation of dust. Hotel Clean and Hospital Clean begin where the construction site ends, i.e., outside the hoarding and are generally done by the staff of the health care setting.

Prior to the construction and renovation activities, an ‘Infection Control (IC) Risk Assessment’ (Appendix A 1, 2, 3) must be completed. The risk assessment consists of the following 3 steps:-

I. Identify the type of construction project. (Appendix A1)
II. Identify those patient areas at risk. (Appendix A2)
III. Match the type of construction activity with the patient risk group. (Appendix A3)

Infection control precautions to be taken for respective class of risks are described in Appendix A3.1 and 3.2

### Recommendations

1. Prior to any construction or renovation activity, patients who are at risk should be identified as high risk, medium risk and low risk patients. [BIII]
2. Pre-construction and renovation consultation should be carried out in advance between all the stakeholders. [BIII]
3. During construction activities, it is necessary to contain or minimize dispersal of dust. [BIII]
4. Once the project is started, the Infection Control Team shall conduct rounds in order to verify infection control compliance. [BIII]
5. If corrective measures are not adequate; the Head of Department of Infection Control has the authority to stop further work on the renovation/construction project until corrective measures are adequately addressed. [CIII]

### Cleaning and sanitation practices in food preparation areas

The manufacturer’s instructions regarding the use and maintenance of equipment as well as the use of chemicals for cleaning and sanitizing food contact surfaces should be followed. Food contact surfaces such as those of sinks, tables, equipment, utensils, thermometers, carts should be washed, rinsed and sanitized before each use, between uses and anytime contamination occurs.

### Recommendations

1. All staff working in food preparation areas should report to work in good health and should practice good personal hygiene. [BIII]
2. The manufacturer's instructions regarding the use and maintenance of kitchen equipment should be followed. [BIII]
3. The manufacturer's instructions regarding the use of chemicals for cleaning and sanitizing food contact surfaces should be followed. [BIII]
4. Cleaning eating utensils using the 3-compartment sink should involve the following steps:
  - Washing with detergent
  - Rinsing with clean detergent
  - Sanitizing by chlorine or hot water [BIII]
5. When cleaning eating utensils using the dishwashing machine, the manufacturer's instructions should be followed for use. [BIII]

### Environmental cleaning after flood

Flood waters are characterized as either clear water, gray water, or black water [17]. Clear water refers to water from tap or rain water, while gray water refer to water from sinks, showers, tubs, and washers. In contrast, black water refers to flood water contaminated with waste from humans and animals [17]. The recommended post-flood cleaning and disinfection processes are contingent upon the type of flood water and the material to be cleaned. A Spaulding classification is generally recommended for cleaning and disinfection for all healthcare equipment [18, 19]. Special approaches for area decontamination may be needed related to use of ultraviolet light C (UVC) and use of hydrogen peroxide vaporizers, in situations where fungal bioburden were higher than acceptable level [20–29].

### Recommendations

1. The recommended post-flood cleaning and disinfection processes are dependent upon the type of flood water and material to be cleaned. [BIII]
2. Cleaning processes for non-medical devices and surfaces should include detergent and water, clean water rinse, use of 10,000 ppm (1 % available chlorine) chlorine-based concentration disinfection, and air dry. An exception is use of 100,000 ppm (10 % available chlorine) chlorine-based disinfection for non-medical devices and surfaces suspected of fungal contamination. [BII]
3. Special approaches (e.g., UVC and use of hydrogen peroxide vaporizers) can be used in situations where fungal bioburden were higher than acceptable level. [BII]
4. No recommendation can be made for other special approaches for area decontamination including use of ozone or other disinfectant (e.g. QUATS).

### Care and storage of cleaning supplies and utility rooms

All chemical cleaning agents and disinfectants should be appropriately labeled and stored in a manner that eliminates risk of contamination, inhalation, skin contact or personal injury. Chemicals must be clearly labeled with Safety Data Sheets (SDS) readily available for each item in case of accidents.

### Recommendations

1. Cleaning agents and disinfectants shall be labeled with SDS information. [CIII]
2. Cleaning agents and disinfectants shall be stored in a safe manner in storage rooms or closets. [CIII]
3. Automated dispensing systems, which are monitored regularly for accurate calibration, are preferred over manual dilution and mixing. [BIII]
4. Disinfectants should be dispensed into clean, dry, appropriately-sized bottles that are clearly labeled and dated; not topped up; and discarded after the expiry date. [AII]
5. Equipment used to clean toilets:
  - Should not be carried from room-to-room;
  - Should be discarded when the patient/resident leaves and as required; and
  - Should minimize splashing. [BIII]
6. Sufficient housekeeping rooms/closets should be provided throughout the facility to maintain a clean and sanitary environment. [BIII]
7. Cleaning and disinfection equipment should be well maintained, in good repair and be cleaned and dried between uses. [BIII]
8. Mop heads should be laundered daily and dried thoroughly before storage. [BIII]
9. Cleaning carts should have a clear separation between clean and soiled items, should never contain personal items and should be thoroughly cleaned at the end of the day. [BII]

### Laundry and bedding

Policies and procedures should address the collection, transport, handling, washing and drying of soiled linen, including protection of staff and hand hygiene. National regulations must be followed if the facility does its own laundry.

### Recommendations

1. If the facility does its own laundry, national laundry regulations must be followed. [CIII]
2. There must be clear separation between clean and dirty laundry. [AII]
3. There must be policies and procedures to ensure that clean laundry is packaged, transported and stored in a manner that will ensure that cleanliness is maintained. [BII]
4. There must be designated areas for storing clean linen. [BII]

### Assessment of cleanliness and quality [30–39]

There are several methods for assessing environmental cleanliness:

- Conventional program of direct and indirect observation (e.g., visual assessment, observation of performance, patient/resident satisfaction surveys);
- Enhanced program of monitoring residual bioburden (e.g., environmental culture, adenosine triphosphate - ATP-bioluminescence); and environmental marking tools (e.g., fluorescent marking)

Environmental marking measures the thoroughness of cleaning using a surrogate marking system. It involves the use of a colorless solution or Glo Germ powder or gel that is applied to objects and surfaces in the patient’s environment prior to cleaning, followed by detection of residual marker (if any) immediately after cleaning, usually involving fluorescence under ultraviolet (UV) light. Environmental marking may be used either on a daily basis to assess routine cleaning, or prior to discharge to assess terminal cleaning.

### Recommendations

1. There should be a process in place to measure the quality of cleaning in the health care setting. [BII]
2. Methods of monitoring cleanliness should include at least the conventional visual assessment and/or fluorescent marking. [BII]
3. Results of cleaning audits should be collated and analyzed with feedback to staff, and an action plan developed to identify and correct deficiencies. [BIII]

### Staff education

Staff education, thus, plays a vital role in meeting these requirements and in educating involved healthcare personnel on various infection control aspects on hospital environmental control and cleaning, particularly in view of rapid staff turnover that occurs at many resource-limited settings.

Management and supervisory staff should receive training and education that also includes chain of infection, pest control, and outbreak response. Informal education should be complemented with in-service education on hand hygiene, appropriate and early diagnosis of infections, indications for area decontamination and hospital cleaning, and isolation precautions and policies [40]. Ongoing staff education is important due to the new research and guidelines published every year, advancements in technology, and regulatory demands. Education should be focused on the role of environmental control to limit the spread of drug-resistant pathogens [41]. Educational campaigns, including facility-wide, unit-targeted, and informal educational interventions, to enhance adherence to infection prevention and control can decrease MDRO transmission [41]. The focus was to encourage a behavior change through improved understanding of the problem MDRO that the facility was trying to control. Whether the desired change involved hand hygiene, antimicrobial prescribing patterns, or something else, enhancing understanding and creating a culture that supported the desired behavior were viewed as essential to success.

### Recommendations

1. All aspects of environmental cleaning must be supervised and performed by knowledgeable, training staff. [BIII]
2. Housekeeping must provide a training program which includes:
  - A written curriculum
  - A mechanism for assessing proficiency
  - Documentation of training and proficiency verification
  - Orientation and continuing education [BIII]
3. Infection prevention and control education provided to staff working in Housekeeping should be developed in collaboration with Infection Prevention and Control and Occupational Health and Safety and must include:
  - The correct and consistent use of routine practices
  - Hand hygiene and basic personal hygiene
  - Signage used to designate Additional Precautions in the health care setting
  - The appropriate use of personal protective equipment
  - Prevention of blood and body fluid exposure, including sharps, safety [BIII]
4. Housekeeping managers and supervisors must receive training and be certified. [BIII]

## Conclusion

We recommend healthcare facilities to include an environmental hygiene program as part of their Infection Control program. The goal of this program is to keep the environment safe for patients, staff and visitors. The best practices set out in the APSIC Guidelines For Environmental Cleaning And Decontamination will provide criteria for cleanliness that may be adopted by Environmental Services managers for their use or for the use of contracted service.

## Appendix A1

**Table 3:** STEP 1: Identify The Infection Control Risk Assessment (ICRA). TYPE OF CONSTRUCTION ACTIVITY OR PROJECT (circle type of project)

**Type A**	**Inspection and non**-**Invasive Activities** Includes but not limited to: • Activities which do not generate dust or require cutting of walls or access to ceilings other than for visual inspection. eg. Removal of ceiling tiles for visual inspection, painting but not sanding, electrical work, minor plumbing that disrupt water supply to localized patient care area (e.g. in one room)
**Type B**	**Small scale short duration activities which create minimal dust** Include but not limited to: eg. Activities that require access to duct spaces, cutting of walls, ceilings, sanding of walls for painting, plumbing that requires disruption to water supply of more than one patient care area (> two rooms) for less than 30 min.
**Type C**	**Work that generates a moderate to high level of dust or requires demolition or removal of any fixed building components or assemblies** Include but not limited to: • Sanding of walls for painting or wall covering • Removal of floor coverings, ceiling tiles and case work • New wall construction • Minor duct work or electrical work above ceilings • Major cabling activity • Any activity that cannot be completed within a single work shift.
**Type D**	**Major demolition**, **construction & renovation projects** Includes but not limited to: • Activities that require consecutive work shifts • Require heavy demolition or removal of a complete cabling system • New construction/New building project.

## Appendix A2

**Table 4:** STEP 2: Using the following Table, identify the patient risk groups affected by the activity. If more than one risk group will be affected, select the higher risk group. RISK GROUPS. IDENTIFY PATIENT AT RISK (circle area involved)

LOW RISK	MEDIUM RISK	HIGH RISK	HIGHER RISK
• All office area • Nonclinical areas	▪ Admitting Unit ▪ Outpatient Areas ▪ Food prep areas ▪ Radiology ▪ Nuclear Medicine ▪ MRI ▪ Endoscopy Unit ▪ Outpatient Physical Therapy (Rehab) ▪ Psychiatric Services (outpatient) ▪ Cardiology services (outpatient)	▪ Trauma & Emergency Department ▪ Labor & Delivery Ward ▪ Pediatrics Wards ▪ Pharmacy ▪ Newborn Nursery ▪ Clinical Pathology ▪ Day Care Surgery ▪ Central Stores ▪ Laboratories ▪ Medical Units ▪ Surgical Units ▪ Hemodialysis Unit	▪ Bone Marrow Transplant Unit ▪ Burn Intensive Care Unit ▪ Cardiac Cath Lab ▪ Pharmacy Sterile Unit ▪ Operating Rooms ▪ Negative Air / Positive Air Pressure Rooms ▪ Isolation Rooms (in all wards/ Units) ▪ Intensive Care Units ▪ Cardiac Intensive Care Unit ▪ Dialysis Unit ▪ PICU ▪ CSSD ▪ Oncology Ward ▪ Any area / ward / unit caring for immunocompromised patients

## Appendix A3

**Table 5:** STEP 3: Match the planned Construction activity type (A,B,C,D) with the Patient Risk Group (low, medium, high, highest) to determine the Class of Precautions (1, 11, 111,1 V) or level of infection control activities required. RISK CLASS DETERMINATION

PATIENT RISK GROUPS	CONSTRUCTION ACTIVITY	CONSTRUCTION ACTIVITY	CONSTRUCTION ACTIVITY	CONSTRUCTION ACTIVITY
	TYPE A	TYPE B	TYPE C	TYPE D
Low Risk	1	11	11	111/ 1 V
Medium Risk	1	11	111 or 11	1 V
High Risk	1	11 or III	111/ 1 V	1 V
Highest Risk	11 or I-III	111/ 1 V	111/ 1 V	1 V

Description of Required Infection Control Precautions by class: Refer Appendix A3.1 and 3.2

**Table 6:** Description of Required Infection Control Precautions by Class

	Pre-Construction	During Construction of Project	Upon Completion of Project
**CLASS 1**		1. Execute work by methods to minimize raising dust from construction operations 2. Immediately replace a ceiling tile displaced for visual inspection	Clean work area upon completion of task.
**CLASS 2**	1. Identify the type of construction project 2. Identify those patients at risk area 3. Discuss with Department of Engineering, Department of Facilities, Department of Nursing, Department of OSHA and Contractor regarding control of dust generation, patient placement and putting up of barriers 4. Get infection control permit	1. Provide active means to prevent airborne dust from dispersing into atmosphere. 2. Water mist for work surfaces to control dust while cutting 3. Seal unused doors with duct tape 4. Block off and seal air vents. 5. Place dust mat at entrance and exit of work area. 6. Remove or isolate air handling system in areas where work is being performed	1. Contain construction waste before transport in tightly covered containers. 2. Wipe work surfaces with detergent and water/disinfectant forward areas. 3. Wet mop and/ or vacuum with HEPA-filtered vacuum before leaving work area. 4. Remove alterations of air handling system in the area where the work is being performed.
**CLASS 3**	1. Identify the type of construction project 2. Identify those patients at risk area 3. Discuss with Department of facilities, Department of Nursing, Department of OSHA and Contractor regarding control of dust generation, patient placement and putting up of barriers 4. Get infection control permit	1. Remove or isolate air handling system in area where work is being done to prevent contamination of duct system. 2. Complete all critical barriers i.e., sheetrock, plywood, plastic, to seal area from non-work area 3. Maintain negative air pressure within work site if necessary. Cease work immediately if negative pressure lost. 4. Contain construction waste before transport in tightly covered containers. 5. Cover transport receptacles or carts. Tape covering unless solid lid.	1. Do not remove barriers from work area until completed project is inspected by the Department of Engineering, Department of Infection Control, and thoroughly cleaned by Department of Facilities. 2. Remove barrier materials carefully to minimize spreading of dirt and debris associated with construction. 3. Vacuum work area including barriers with HEPA-filtered vacuums. 4. Wet mop area with water & detergent /disinfectant in ward areas. 5. Remove alteration to the air handling system in areas where work is being performed.
**CLASS 4**	1. Identify the type of construction project 2. Identify those patients at risk area 3. Discuss with Department of Facilities Department of Nursing and Department of OSHA and Contractor regarding control of dust generation, placement of patients and putting up of barriers 4. Get infection control permit	1. Isolate air handling system in area where work is being done to prevent contamination of duct system. 2. Complete all critical barriers i.e., sheetrock, plywood, plastic, to seal area from non-work area 3. Maintain Negative air pressure within the work site. Cease work immediately if negative pressure lost. 4. Contain construction waste before transport in tightly covered containers. 5. Cover transport receptacles or carts. Tape covering unless solid lid. 6. Seal holes, pipes, conduits and punctures appropriately. 7. Construct anteroom and require all personnel to pass through this room so they can be vacuumed using HEPA vacuum before leaving the work site; or they can wear cloth or paper coveralls that are removed each time they leave the work site 8. All personal entering the work site are required to wear shoe covers. Shoe covers must be changed each time the worker exits the work area.	1. Do not remove barriers from work area until completed project is inspected by Department of Engineering, Department of Infection Control and thoroughly cleaned by Department of Facilities 2. Remove barrier materials carefully to minimize spreading of dirt and debris associated with construction. 3. Contain construction waste before transport in tightly covered containers. 4. Contain transport receptacles or carts. Tape covering unless solid lid. 5. Vacuum work area with HEPA-filtered vacuums. 6. Wet mop area with disinfectant. 7. Remove isolation of air handling system in areas where work was performed.
	Pre-Construction	During Construction Project	Upon Completion Project
**CLASS 2**, **3 & 4**			1. Put on air conditioning full blast for 2 days 2. Lock doors to prevent intruder 3. Final walk through inspection 4. Observe if any dust on furniture 5. Review effectiveness of any problems noted before 6. Air sampling if necessary.
